# Review of "Evaluating Clinical Research – All that glitters is not gold" by Bengt D. Furberg and Curt D. Furberg

**DOI:** 10.1186/1745-6215-9-26

**Published:** 2008-05-09

**Authors:** Desmond Julian

**Affiliations:** 1 7 Netherhall Gardens, London, NW3 5RN, UK

## 

Doctors prescribe inefficiently. Many conditions, such as hypertension, are inadequately treated whereas others, such as depression, are overtreated. Bearing in mind the plethora of guidelines now available on virtually every sector of medicine, this seems inexcusable. Whether due to ignorance, indolence, or incredulity, many physicians are not implementing the recommendations of 'experts'.

Conscientious clinicians may think that the advice available from the 'experts' does not apply to the patients they see and will want to assess the evidence for themselves, so that their practice will provide the best management for their patients. It is for such people that this book has been written. The writing is excellent and it is embellished by amusing cartoons and witty one-line quotations. The authors have tried, but not completely succeeded, in avoiding statistics. Their extensive personal experience has enabled them to give good examples to illustrate their points, although they also quote widely from the world literature.

They start by emphasizing the importance of evaluating the balance between benefit and harm. Unfortunately, all too often, papers and presentations, as well as pharmaceutical companies, often highlight the benefits of new treatments while saying little about the adverse effects.

The value of randomized controlled trials (RCTs) as the bedrock of evidence-based medicine is unquestionable, but the shortcomings are often ignored-especially by 'experts'. The authors discuss both the strengths and weaknesses of randomized RCTs. The chapter on weaknesses is largely concerned with the inability of RCTs, because of their limited size, to recognize rare side effects. Although referred to later in the book, the unrepresentative nature of those recruited into trials is given insufficient attention. This is a major reason why practising physicians are often sceptical about the results of trials.

Having stressed that RCTs 'rank highly in terms of reliability for evaluating treatments,' one may question the authors' claim that 'at the top of the ranking are meta-analyses of clinical trials'. In fact, as experience has shown, large individual trials may provide more convincing evidence than a meta-analysis.

In later chapters, they consider such important topics as the doubtful value of surrogate endpoints and biochemical markers, and they also discuss whether active controls can be relied upon and whether all drugs in a class are interchangeable. Much attention is paid to the measurement of adverse drug reactions but here, as elsewhere, discussion is focused on practices in the United States, whereas these issues are often better tackled in other countries (where many of their readers will be located).

Overall, this is an excellent book that can be strongly recommended to the target audience which is, as the authors state, ''clinicians and others in the health care field as well as employees in companies manufacturing drugs, devices and other medical products'.

## Competing interests

The author declares that he has no competing interests.

